# The Influence of Media in Purchasing Decisions for Recycled Construction and Demolition Waste Products: An Functional Near Infrared Spectroscopy Study

**DOI:** 10.3389/fnins.2022.881537

**Published:** 2022-05-26

**Authors:** Zhikun Ding, Zhiyu Zhang, Weilin Chen

**Affiliations:** ^1^Key Laboratory for Resilient Infrastructures of Coastal Cities, Shenzhen University, Ministry of Education, Shenzhen, China; ^2^Guangdong Laboratory of Artificial Intelligence and Digital Economy (SZ), Shenzhen, China; ^3^Sino-Australia Joint Research Center in BIM and Smart Construction, Shenzhen University, Shenzhen, China; ^4^Shenzhen Key Laboratory of Green, Efficient and Intelligent Construction of Underground Metro Station, University of Shenzhen, Shenzhen, China

**Keywords:** purchase decision, functional near-infrared spectroscopy (fNIRS), construction and demolition (C&D) waste recycling products, prefrontal cortex, media

## Abstract

The increasing hazards caused by construction and demolition (C&D) waste is an inevitable problem in the development of the construction industry. Many countries have successively launched many policies to encourage and guide the recycling of C&D waste, which has greatly improved the recycling rate of C&D waste. However, most of these policies only regulate contractors but do not promote C&D waste recycling products enough. It has led to an increase in the production of C&D waste recycling products while the acceptance in the market is generally low. Consumers believe that products made with “garbage” may have problems such as quality defects. In order to explore a measure that can mitigate this problem, this study uses functional near infrared spectroscopy (fNIRS) to investigate whether the influence of media can increase consumers’ willingness to purchase products for recycling construction and demolition waste, and thus increase consumers’ choice to purchase products for C&D recycling waste. This experiment consists of two phases. First, a pre-test experiment to obtain pre-intervention brain images characterizing consumers’ original attitudes toward C&D recycling waste products through a functional near-infrared imaging brain technique and a questionnaire. Second, The post-test builds on the pre-test to investigate the effectiveness of the intervention. The activation mechanism of the consumer purchase decision is further investigated by fNIRS data. The behavioral results showed that the choice of recycled C&D waste products was significantly higher after the intervention. The fNIRS results further revealed the significantly higher activation of the dorsolateral prefrontal cortex (dlPFC), orbitofrontal cortex (OFC), and medial prefrontal cortex (mPFC) after the intervention. These findings suggest that consumers’ purchase willingness is significantly improved after intervention, and their purchase behavior changed substantially. This study also demonstrates the great potential of fNIRS for interdisciplinary research in engineering management and neuroscience.

## Introduction

Construction and demolition (C&D) waste accounts for about 36% of the total global waste generation each year, causing serious environmental pollution and posing a public health hazard (Chinda, 2016; UPLRCSM, 2021). To reduce the negative impact of C&D waste, stakeholders should reduce its generation as much as possible and increase its recycling (Tam et al., 2018). It is estimated that 75% of C&D waste ends up in landfills. Countries such as Japan, the United Kingdom and the Netherlands can recycle nearly 80% of their C&D waste, but many more countries, such as Italy and China, have much lower recycling rates (Duan et al., 2019). Market adoption of recycled C&D waste is slow because of quality and price (Ma et al., 2022). The attitude of contractors can have an impact on C&D waste management. In the absence of government regulation, the criteria for consumer choice depends on the economic cost, even though using C&D recycling waste have a more positive impact (Al-Sari et al., 2012). It is difficult to have a price advantage compared to natural construction materials due to the high cost of research and development, production, transportation, and marketing (Wu et al., 2022). In addition, consumers have limited knowledge of recycled products and have doubts about their quality, leading to low recognition of recycled products in the market (Ruirui, 2020). There is also an emotional reluctance to accept these recycled products and a subjective perception that recycled products are “unsafe,” “unreliable” or even “harmful,” further limiting the development of the recycling industry (Liu et al., 2019). The lack of publicity about recycling products constrains consumers’ knowledge of their products and makes them feel suspicious, which in turn weakens the awareness of all parties involved in recycling (Ye, 2014; Cao et al., 2021). It is difficult to make a huge breakthrough in the development of the production processes of recycled products in a short period, and changing consumer perceptions is decisive for the construction of the recycled industry chain (Yang, 2017). Therefore, there is a need to strengthen the promotion of C&D waste recycling products to raise consumers’ awareness of recycling and make positive purchasing decisions.

The development of media technology has facilitated consumers’ access to market information and has brought benefits to product marketing (Wang et al., 2018). With the improvement of C&D waste recycling technology and the quality standardization, recycled products have been able to produce qualified goods and in some areas even better substitutes for natural products. In practice, the C&D waste recycling rate is still low. This is because neither the government nor the recycling companies use the media to promote recycled products. The influence of the media weakens the stereotypes about recycled C&D waste products, allowing consumers to make more rational purchasing decisions. Although the effectiveness of this intervention strategy has been demonstrated in many studies, it is also challenged by some scholars (Wang et al., 2018). Therefore, it is essential to further explore the influence of media on consumers’ purchasing decisions.

The purchase decision is a pattern of consumer behavior in which consumers identify and follow various stages of decisions to complete their choices. As the study of consumer buying behavior has intensified, some literature have studied the relationship between attitudes, imagery, and behavior, and some theoretical models have been built. The Theory of Reason Action (TRA), Theory of Planned Behavior (TPB), and Technology Adoption Model (TAM) is widely accepted. These three theories are commonly used to analyze the behavioral path associations of consumers. They capture the factors that influence consumer behavior and the interrelationship between these factors. First proposed by Ajzen and Fishbein, the TRA assumes that each individual behaves rationally. It predicts behavior by measuring beliefs, attitudes and intentions, demonstrating that psychological factors underlie volitional behavior (Woo et al., 2015). TPB was initially an important theory for predicting human social behavior, and in recent years has begun to appear in marketing for analyzing and predicting rational consumer behavior (Ahmed Shaikh et al., 2020). TAM was based on the theory of rational behavior and the theory of planned behavior. The theory argues that individual behavior is determined by behavioral intentions, which are jointly determined by an individual’s attitudes and perceived usefulness (Douglass, 1977).

Consumers are not fully rational when making decisions; they rely on considering maximum utility and also on irrational factors, such as emotions. Therefore, changes in research methods have provided new ideas for researchers and marketers to understand consumer decisions and behavior. Traditional surveys collect data relevant to consumer decisions that are not objective enough. Under the influence of social opinion, people tend to portray a positive self-image in their self-reports, making themselves appear environmentally conscious. This social approval effect may cause consumers to unconsciously or consciously conceal their true preferences for products during a shopping process, influencing the data collected by researchers in self-report (Ajzen, 1987). Researchers have also realized that direct measurement methods such as questionnaires and interviews cannot prevent participants from making perverse decisions during the study, due to excessive speculation about the true purpose of the experiment (Davis, 1989; Pop et al., 2021). This has led to methods combined with cognitive neuroscience that can help explain the underlying mechanisms and help researchers better understand consumer preferences (Moskowitz and Carter, 2018). Researchers can use brain imaging to measure consumers’ neural activity to more accurately explain consumer motivation and the effects of intervention strategies during the purchase process (Knutson and Bossaerts, 2007; Venkatraman et al., 2014).

fNIRS is an emerging non-invasive neuroimaging technique. It reflects the activation state of brain regions by monitoring the relative changes in local oxygenation in the brain (Bruderer Enzler et al., 2019). Compared to other neuroimaging methods such as functional magnetic resonance imaging (fMRI) and Electroencephalogram (EEG), fNIRS is portable, motion tolerant, and safe. It also has a relatively high temporal and spatial resolution and has been widely used in recent years as a tool for monitoring brain activity. Few studies have used fNIRS to study consumers’ willingness to purchase recycled C&D waste products. Human economic decision making is typically seen as a rational, cognitive process. The prefrontal cortex, a higher cognitive area of the brain, is often thought to be closely associated with consumer purchase decisions and willingness to pay (Raichle and Mintun, 2006; Bakardjieva and Kimmel, 2016). Therefore, this study will use fNIRS to explore the impact of media intervention strategies on consumer purchase decisions for recycled C&D waste products at the neural dimension. The prefrontal cortex activation patterns will be discussed to explain the reasons for the differences that arise before and after the intervention.

## Materials and Methods

### Neurovascular Coupling Mechanism

The brain, as the most central organ in the human central nervous system, although its mass occupies only about 2% of the body weight, energy consumption occupies about 20% of the total energy consumption of the human body (Knutson et al., 2007). It requires large amounts of oxygen and glucose to produce enough energy to maintain normal operation, and the higher the intensity of brain activity, the more energy will be consumed. The Italian physiologist Mosso (Ramsoy et al., 2018) first reported this physiological phenomenon in 1881. He measured changes in brain activity in the right prefrontal cortex of a patient with craniosynostosis and observed a significant increase in arterial activity in the right prefrontal cortex when the patient started performing computational tasks. This phenomenon arises spontaneously from the increased intensity of brain activity, which is known as neurovascular coupling (Clarke and Sokoloff, 1999) (Neurovascular Coupling; NVC).

The mechanism of neurovascular coupling is determined by the physiological structure of the brain. When the brain responds to some external stimulus, it activates the brain area responsible for processing the corresponding information. The energy demand of this area rises for a short period, but the oxygen exchange rate between the blood vessels in the brain and the brain is fixed, which leads to the need for the blood vessels to increase the flow of blood to ensure oxygen sufficiency. At the same time, the efficiency of glucose catabolism is further increased when there is a large activation of neurons, i.e., more energy can be produced per unit of glucose. The demand per unit of oxygen is also reduced for the same energy requirement. With the combined effect of these two mechanisms, there is an excess of oxygen, an increase in the concentration of oxygenated hemoglobin, and a decrease in the concentration of deoxygenated hemoglobin in the activation zone (Bruderer Enzler et al., 2019). Thus, local activation in the brain can be characterized to some extent when there is a local increase in oxyhemoglobin concentration and a decrease in deoxyhemoglobin concentration in the brain. Such changes can be detected by near-infrared light, enabling non-invasive monitoring of brain activity.

### Functional Near Infrared Spectroscopy Data Analysis Principles

The principle of the fNIRS technique can be summarized as follows: using the good tissue penetration and absorption properties of NIR light, combined with the modified Lambert-Beer law, we can infer the changes in oxygenated (HbO) concentration and the deoxygenated (HbR) concentrations over a specific period, and thus infer the activity state of the brain. In this inference process, a key “bridge” is missing the correlation between hemoglobin concentration change and cognitive activity. Therefore, effective monitoring of the cognitive activity by fNIRS relies on the Hemodynamic Response Function (HRF).

**FIGURE 1:**
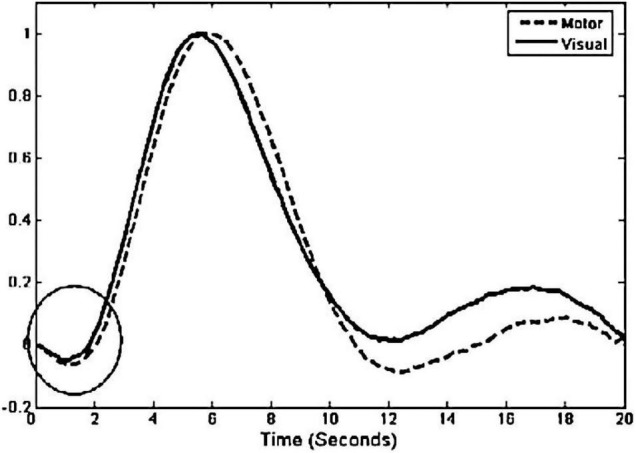
HRF experience curve (Mosso, 1881).

The HRF represents the relationship between the experimental task and changes in hemoglobin concentration. Figure 1 illustrates the empirical curve of the HRF. The curves in the figure show that after being stimulated by the task, the local brain starts to respond. However, at the beginning of the brain activity, the lack of local supply causes a brief decrease in HbO concentration until about 2 s after the stimulus onset, when the blood brought by the neurovascular coupling mechanism allows the HbO concentration to begin to rise and reach a peak within 5–8 s. If no other stimulation conditions are added during this period, the HbO concentration gradually decreases to a level at or below baseline after the peak; the whole process of signal change lasts about 10 s and reflects the form of cerebral blood flow in response to the stimulation event.

To further localize the brain regions responsible for a specific event for analysis, the most commonly used approach is the General Linear Model (GLM), which divides the signal collected by the detector into two components: one part is interpretable and associated with the stimulus event, the other part is not interpretable and is residual in the model. That is:


Y=X⁢β+ε

where Y is the signal measured by the fNIRS device, a matrix composed of the explanatory variables. x is the design matrix, which contains two types of explanatory variables: one is information related to the task design, a function obtained by convolving the stimulus sequence with the HRF, the change curve that should be produced by hemoglobin in the purely theoretical state; the other is non-random noise that would interfere with the non-random noise of the fNIRS signal, such as task-irrelevant physiological activity. β is a model parameter representing the degree of explanation of the corresponding explanatory variables. ε_*i*_ is the residuals in the model that cannot be explained. Estimation of the parameters β and residuals ε based on the least squares method yields.


$$\hat{\beta }={\left(X^{T}\,X\right)}^{-1}\,X^{T}\,Y$$

$$\hat{\varepsilon }=Y-\hat{Y}=Y-X\,\hat{\beta }=\left(I-X\,{\left(X^{T}\,X\right)}^{-1}\,X^{T}\right)\,Y$$

Assuming the existence of two experimental tasks and 18 probe channels, $\hat{\beta }$ is an 18*2 matrix, and a β-value exists for each channel in each experimental condition corresponding to the degree of activation of the brain regions measured by the different channels in that experimental condition. And in order to extract the experimental effects for a specific task (e.g., extract ${\hat{\beta }}_{1}$), a contrast vector c needs to be constructed.


$$c^{T}=\left[1 0\,\ldots \,0\right]$$

$$c^{T}\,\hat{\beta }={\hat{\beta }}_{1}$$

After obtaining the activation levels at the individual level, an appropriate *t*-test model should be selected to verify the presence of false positive errors according to the experimental design. A significance threshold of = 5% is used. When the inter-cluster activation level of a channel exceeds the significance threshold, it means that the brain region corresponding to that channel is significantly activated during the experiment and that the brain region is highly relevant to the experimental task.

## Data Collection and Analysis

### Participants

In experimental research, internal validity is the evaluation of the authenticity of experimental results and is the basic guarantee of external validity. An experiment with low internal validity will not guarantee the authenticity of its results or generalize the results to other settings. Therefore, priority should be given to safeguarding the internal validity of experiments in experimental design (Siming et al., 2019). t is generally required to select experimental participants that are close in age and education in order to obtain high internal validity. Therefore, most of the current studies selected university students as the participants. In this study, 22 university students from Shenzhen University were recruited to participate in the experiment (22.5 ± 3.5 years of age, 12 males, and 10 females). This sample consisted of 8 undergraduate students and 14 postgraduate students. All participants are right-handed with normal or corrected vision, and do not have any history of neurological or psychiatric disorders. Information about and calls for participation in the study are published on the bulletin board system of the campus network. Every participant received ¥70 CNY (about $11 USD) for the participation. All participants completed a written informed consent form in compliance with the Declaration of Helsinki prior to the experiment. The study protocol was approved by the Institutional Review Board at Sino-Australia Joint Research Center in BIM and Smart Construction, Shenzhen University.

### Experimental Task

**FIGURE 2:**
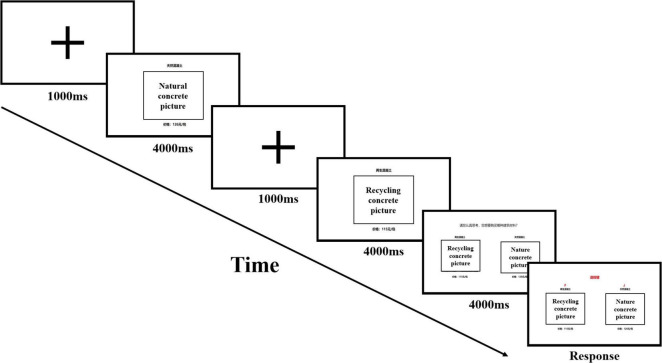
Experimental procedures.

**FIGURE 3:**
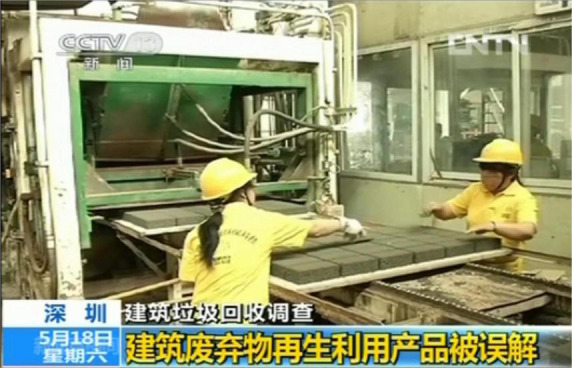
Sample video screenshot. Source: China Internet TV.

The experimental task aimed to test whether publicity through the media could lead to a larger preference for recycled C&D waste products among the participants. The mobile fNIRS measurement device was used during the experiment. The experiments were arranged in a quiet laboratory, and a signboard was placed outside the door to avoid possible external disturbances. Before the formal experiment, participants were required to fill in a questionnaire to identify their level of awareness and outward attitudes toward recycled C&D waste products. The experiment was divided into two parts. Before the media intervention, the participants were presented with 24 different scenarios of construction material purchase decisions. Each scenario contained a picture of a natural construction material product, a picture of a recycled C&D waste product and their corresponding prices. They were asked to make a purchase decision in each scenario (Figure 2). At the end of the first part of the experiment, the participants were asked to fill out an explicit attitude questionnaire and watch a 5-min news video from China Central Television (CCTV) News Channel. The video illustrated the benefits of recycled C&D waste products in terms of quality, durability, and environmental friendliness, including a case study from the participants’ city (Shenzhen). The sample screenshot of this video is shown in Figure 3. At the end of the video, participants were asked to answer several questions related to the video, all of which were answered correctly before the experiment could continue. To control for variables, the same procedures were used before and after the intervention, and a 2 × 12 block design was used for both parts of the experiment. At the end of the experiment, participants were required to fill out the explicit attitude questionnaire again.

As fNIRS detection of brain activity relies on a vascular coupling mechanism, it cannot respond to stimuli in a short period. The short duration of a single stimulus and the low intensity makes it difficult to stimulate brain activity sufficiently. Therefore, the experiment was divided into 12 blocks following the block design method. Each block consisted of 2 product information stimuli, with the effects of each stimulus gradually superimposed to reinforce the experimental effects and better detect activated brain areas. Then, at the end of each step there was a rest period lasting 30 s to allow the hemoglobin concentration to return to normal levels.

### Functional Near Infrared Spectroscopy Data Collection

**FIGURE 4:**
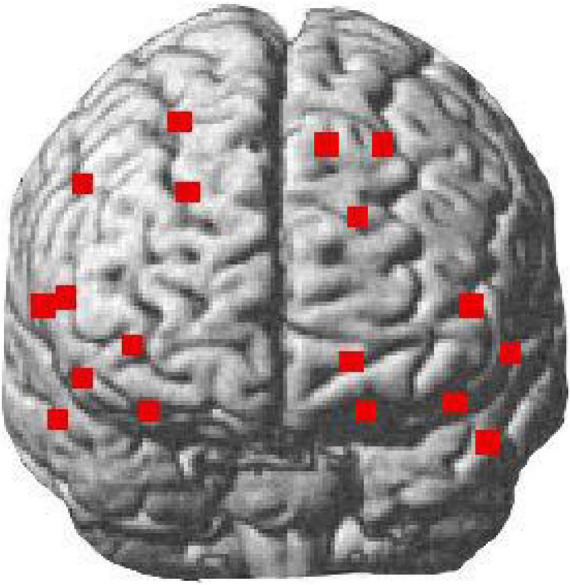
The configuration of the fNIRS channels. The dots on the brain model indicate the location of the arranged channels.

The experimental program was prepared using E-prime 3.0 and presented on a 27 inches Dell computer screen (resolution 1,920 × 1,080, refresh rate 60 Hz). A Brite 24 near infrared optical imaging device from Artinis of the Netherlands was used in the experiment. Near infrared light at wavelengths of 762 and 841 nm was used to monitor changes in the HbO and the HbR concentration signals in the participant’s prefrontal lobe. The device contains two sets of sensors, each consisting of five near infrared light emitters and four detectors, creating a maximum of 27 measurement channels. Based on existing studies, a 2 × 7+2 × 2 arrangement was chosen for this experiment, distributed in the prefrontal cortex, frontopolar region, and inferior frontal gyrus of the brain, forming a total of 18 measurement channels. The spatial arrangement is slightly different from the international 10–20 system used in EEG studies. Therefore, we used the 3D head localizer Polhemus Patriot to determine the relative position of each head reference point and probe to obtain the relative spatial position of the 18 probes in the participant’s head. The spatial distribution of all the probes was then converted into the positions of the 18 near infrared measurement channels in the standard spatial coordinates of the Montreal Neurological Institute (MNI) using the Oxysoft software from Artinis. Finally, using the MATLAB based NIRS_SPM plug-in, the positions of the channels can be paired to a standard brain model. The exact arrangement is shown in Figure 4.

### Functional Near Infrared Spectroscopy Data Analysis

**FIGURE 5:**
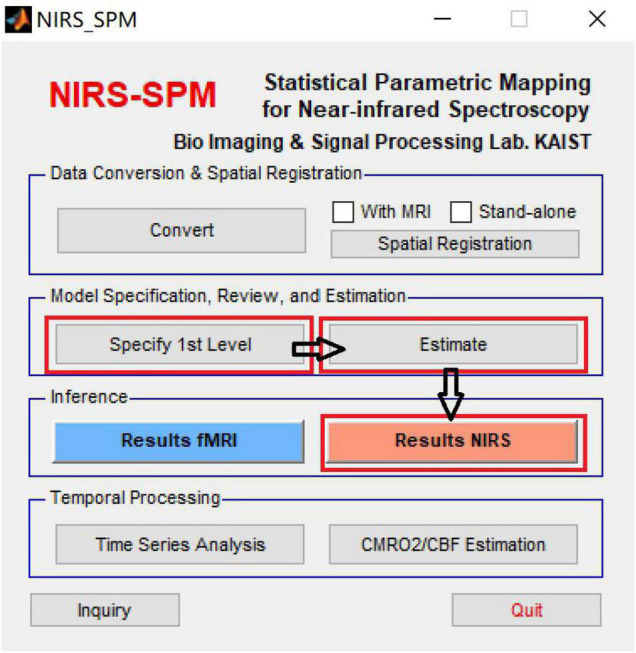
NIRS_SPM plug-in running interface.

The collected light intensity variation data were converted to the HbO and the HbR signals variation data by fNIRS acquisition software Oxysoft, and then the data was analyzed using the NIRS_SPM plug-in after converting the data format to the format required via MATLAB (Figure 5; Lindquist, 2008). Firstly, the Wavelet-MDL algorithm was used to remove noise and data drift due to respiration, heartbeat, and head movement. Then the HRF low-pass filter was used to remove noise from electromagnetic interference. Finally, the design matrix was integrated with the HbO concentration and the HbR concentration signals and a general linear model (GLM) was used to estimate the beta values for each channel under different conditions. Only the HbO concentration signal was used to analyze because of its high signal-to-noise ratio (Druckman et al., 2011).

After the above processing, β-values for each participant were obtained for 18 channels before and after the intervention. The obtained beta values were subjected to a paired samples *t*-test to verify the differences in activation of brain regions before and after the intervention. In addition, to avoid high false positive rates in multiple testing, the significance was corrected by using the False Discovery Rate (FDR) after calculating the significance for each channel (*a* = 0.05).

## Results

### Questionnaire Results

In the semantic difference scale, the participants rated 10 pairs of words on a total of 5 levels from 1 to 5, which were derived from people’s attitudes toward C&D waste recycling products that are commonly found in related literature and online materials. The higher scores indicate that participants were more accepting of C&D waste recycling products.

The scores of each question item before and after the intervention were compared by a one-sample *t*-test, with a score of 3 representing neutrality. The pre-intervention results showed that the scores of all items were not significant except for environmental friendliness and replicability, which were significantly greater than 3. This indicates that the participants’ attitudes toward C&D waste recycling products are neutral. After the intervention, all the items were significantly greater than 3, indicating that the participants were generally in agreement with the C&D waste recycling products after the video intervention. To verify the effect of the video intervention, a paired samples *t*-test was conducted on the pooled data to obtain a significant increase in the score from 3.27 to 4. The intervention format selected for this study significantly improved participants’ subjective evaluations of C&D waste recycling products.

### Behavioral Results

**FIGURE 6:**
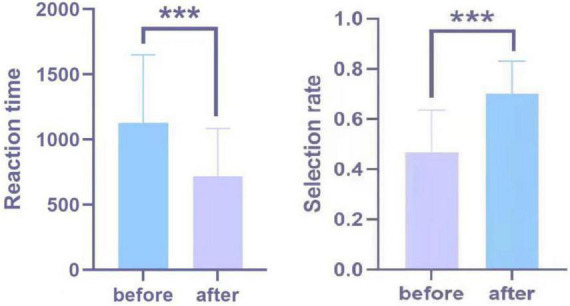
Behavioral data significance graph. The asterisks indicate statistically significant differences (***0.001).

When the stimulus material was presented during the experiment, participants used a button to choose whether to purchase the recycled C&D waste products. The results of the behavioral data analysis consisted of two components: selection rate and response time. A paired-samples *t*-test was conducted on the product selection rate of recycled C&D waste before and after the intervention. The results show that the selection rate of recycling products after the intervention was significantly higher than before the intervention. There were no statistically significant differences in selection rates between genders. Reaction time refers to the minimum time that a consumer has to make a purchase decision after certain cognitive processing. The pre-intervention reaction time was smaller than the post-intervention reaction time. A paired samples *t*-test of the pre- and post-intervention reaction times showed that the post-intervention reaction times were significantly smaller than the pre-intervention reaction times (Figure 6).

### Functional Near Infrared Spectroscopy Results

**FIGURE 7:**
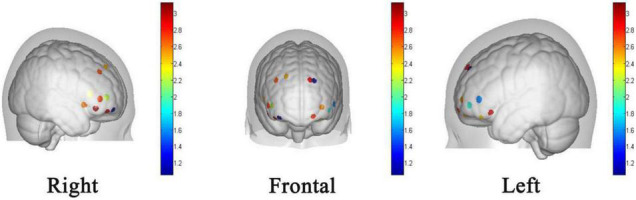
The group level activation t maps derived from the HbO signal. The dots on the brain model indicate the location of the arranged channels, and darker dots indicate higher levels of activation.

Table 1 shows the results of paired sample *t*-test for the corresponding β-values before and after the intervention. Some of the channels produced significant activation of HbO signals after the intervention. The activated areas include the dorsolateral prefrontal cortex (dlPFC) (BA 46, channel 1, channel 5, and channel 7), the medial prefrontal cortex (mPFC) (BA 9, channel 2, and channel 10) and the left orbitofrontal cortex (OFC) (BA 11, channel 13 and 17) (Figure 7). The most significant channel of these activations is channel 10, which is located on the left mPFC. All of these seven channels are located in the prefrontal lobe of the brain. That is, the activation of the prefrontal lobe of the brain was more pronounced after the participants watched the video (Table 2).

**TABLE 1:** Questionnaire scoring.

Title	Before the intervention				After the intervention			
	Mean (M1)	Standard deviation (SD1)	*t*-value	Sig.	Mean (M1)	Standard deviation (SD1)	*t*-value	Sig.
Danger—safety	3.14	0.64	1.000	0.329	4.08	0.58	9.094	0.000
Pollution—environmental	4.14	0.94	5.665	0.000	4.63	0.77	10.343	0.000
Dirty—neat	3.45	0.86	2.485	0.021	4.08	0.78	6.843	0.000
Harmful—helpful	3.5	0.91	2.569	0.018	4.33	0.64	10.254	0.000
Non-promotable—promotable	3.82	0.91	4.231	0.000	4.63	0.58	13.826	0.000
Dislike—like	3.18	0.66	1.283	0.213	3.79	0.66	5.894	0.000
Exclusion—acceptance	3.32	1.04	1.433	0.167	4.04	0.81	6.328	0.000
Doubt—trust	3.09	1.04	1.433	0.167	3.92	0.65	6.868	0.000
No support—support	3.45	0.87	0.491	0.628	4.25	0.67	9.063	0.000
Reluctant to buy—willing to buy	3.27	0.77	1.667	0.110	4	0.72	6.782	0.000

**TABLE 2:** Brain activations.

Channel	Region	BA	*t*-value	Significance
1	R dorsolateral prefrontal cortex	46	2.700	0.013*
2	R medial prefrontal cortex	9	2.491	0.021*
5	R dorsolateral prefrontal cortex	46	2.209	0.038*
7	R dorsolateral prefrontal cortex	46	2.709	0.013*
10	L medial prefrontal cortex	9	2.859	0.009**
13	L orbitofrontal cortex	11	2.761	0.012*
17	L orbitofrontal cortex	11	2.529	0.020*

*0.1, **0.01.

## Discussion

In this study, participants declared on the experimental registration form that they had no exposure to recycled C&D waste products. However, they had negative perceptions about the raw materials, the production process and the quality of the final product. Consumers have a preconceived notion that products made from C&D waste will not meet normal use standards, which will reduce their willingness to purchase. The questionnaire showed that the participants’ attitudes toward C&D recycling waste products were neutral before the intervention. After the video intervention, their attitudes changed to positive. The results of the behavioral data also reflect a significant increase in the selection rate of C&D recycling waste products after the intervention. The results from the NIR also showed a significant increase in the activation of the three regions of dlPFC, mPFC, and OFC.

Making a purchase decision is a complex process, accompanied by psychological and emotional factors. The environmental attributes of recycled C&D waste products mentioned in videos may evoke a sense of environmental ethics in consumers, thus inducing guilty (Nakamura et al., 2021). This negative emotion may further affect cognitive information processing and subsequent decision making (Tong et al., 2011). The dlPFC involves many neuropsychological functions, such as executive functions (planning, action, etc.) (Rees et al., 2014), attention (Lerner et al., 2015), and working memory (Fassbender et al., 2004). The dlPFC is involved in coding moral emotions, so guilty emotions also cause activation in this part of the brain region. A meta-analysis showed that the mPFC was greater associated with emotional processes, while the dlPFC was more active in cognitive tasks (Plassmann et al., 2007). The activation of the dIPFC was higher in participants after the intervention. It indicates that the neural activity of dIPFC, which is closely related to cognitive functions, was enhanced when they were making purchase decisions about C&D recycling waste products.

In the communication with the participants after the experiment, we learned that the participants felt guilty for their prejudices after watching the video. According to previous fMRI studies, OFC is activated by emotional rewards, sensory stimuli and arbitrary reinforcers such as money (Toepper et al., 2010). OFC activation is also involved in coding the willingness to pay, and the stronger the willingness to pay, the higher the activation (Steele and Lawrie, 2004). Many consumer neuroscience studies have shown that mPFC is involved in the purchase decision process, where consumers assess product preferences and values, and can act as a purchase predictor. The mPFC activation showed a significant positive correlation with price differences, suggesting that this brain region is involved in the price assessment process (O’Doherty et al., 2001). Since this study explores the purchase decision, this process usually involves consumers’ consideration of price factors. After better understanding C&D waste recycling products, the price factor becomes progressively more important in the decision-making process. Thus, the neural activity of OFC and mPFC, which are closely related to money or price evaluation, was enhanced after the intervention. Purchase intention is a type of behavioral intention that measures the likelihood that a consumer will purchase a certain product; the stronger the purchase intention, the more likely the purchase will be (Druckman et al., 2011). Thus, greater activation of OFC after the intervention indicated that participants were subjectively more willing to purchase C&D recycling waste products, which ultimately guided their purchase behavior.

We use a neuromarketing approach to better understand the complex phenomena involved in the purchase decision process of consumers of construction materials products and also to provide a more comprehensive assessment of the effectiveness of news media campaigns as an intervention. However, several limitations should be improved in future research. First, participants’ emotions were not considered which may play an important role in the consumer purchase decision process. Second, the sample size could be expanded to further verify the current findings. Thirdly, compared to fMRI, the fNIRS was unable to measure deeper areas of the brain. And due to the limited number of probes, the entire brain could not be analyzed, leaving the study to focus more on the prefrontal cortex.

In summary, this study reveals the neural mechanisms by which consumers purchase recycled C&D waste products and the effectiveness of using media to promote intervention strategies. The feasibility of using fNIRS to study the neural mechanisms of purchase decisions for recycled C&D waste products is preliminarily demonstrated.

## Conclusion

The decision to purchase recycled C&D waste products is an important part of the C&D waste recycling process. A virtual purchase scenario was simulated to measure changes in consumer behavior and neural responses before and after a media intervention. The aim was to investigate whether the influence of media could increase the willingness of consumers to purchase recycled C&D waste products. Traditional research methods such as questionnaires and interviews are more subjective, therefore this study used fNIRS to explore evidence of changes in consumer decision making. fNIRS results further revealed the effects on participants’ prefrontal neural activity after watching a video on recycled C&D waste products. The results of the participants’ behavioral data were cross-validated with the results of the fNIRS data to derive the neural mechanisms underlying the changes in participants’ purchase intentions.

The main findings of this study are as follows:

(1) Media can significantly influence consumer perceptions and purchase decisions. Trust is the basis for influencing consumers’ purchasing decisions throughout the buying process (De Martino et al., 2009). Consumers choose to trust the affirmation of the quality and environmental attributes of recycled C&D waste products that appear in the videos based on their trust in official news. It improves the selection rate and reduces the time spent thinking during the selection process.

(2) Consumers automatically allocate more attention resources to cognition after watching a media video. The right dlPFC was more significantly activated after watching the video. This area is highly correlated with cognitive conflict activity in the brain, suggesting that consumers’ original cognition is altered and they are willing to spend more attentional resources on cognitive processes.

(3) Consumers can induce stronger payment emotions after watching media videos. The OFC produced a more pronounced activation after the consumer watched the video. The OFC was closely related to willingness to pay processing. The higher the activation, the stronger the willingness to pay. It may show that consumers’ willingness to purchase recycled products increased after a clearer understanding of them.

(4) Consumers’ concerns about price increased after watching the video. The mPFC activation suggests that consumers’ concerns about price differences increased after the intervention, possibly because consumers had concerns about the quality of recycled products before the intervention and did not give more consideration to the price factors. The increased trust in recycled demolition waste products after viewing the video led to the price factor being used as a measure in the purchase decision as well.

## Data Availability Statement

The raw data supporting the conclusions of this article will be made available by the authors, without undue reservation.

## Ethics Statement

The studies involving human participants were reviewed and approved by the Sino-Australia Joint Research Center in BIM and Smart Construction, Shenzhen University, Shenzhen, China. The patients/participants provided their written informed consent to participate in this study.

## Author Contributions

ZD and ZZ designed the research and drafted the work. ZZ and WC performed the experiments. ZZ analyzed the data. All authors contributed to the article and approved the submitted version.

## Conflict of Interest

The authors declare that the research was conducted in the absence of any commercial or financial relationships that could be construed as a potential conflict of interest.

## Publisher’s Note

All claims expressed in this article are solely those of the authors and do not necessarily represent those of their affiliated organizations, or those of the publisher, the editors and the reviewers. Any product that may be evaluated in this article, or claim that may be made by its manufacturer, is not guaranteed or endorsed by the publisher.
